# Retraction: Placenta Growth Factor-1 Exerts Time-Dependent Stabilization of Adherens Junctions Following VEGF-Induced Vascular Permeability

**DOI:** 10.1371/journal.pone.0210983

**Published:** 2019-01-16

**Authors:** The PLOS ONE Editors

After publication of this article [1], concerns were raised about the following figure panels:

- Fig 4: The Claudin-5 and Merged images shown for VEGF A (24 hr) appear to duplicate the panels representing PlGF-1 6hr after VEGF A (24 hr).
- Fig 7e: There appear to be vertical discontinuities before lanes 2, 3 and 5 of the Anti-VE-cadherin Y731 (Membrane) immunoblot.
- Fig 8a: In the VE-Cadherin blot, there are background discrepancies in lanes 1–10 versus lanes 11–13, and it appears there may be a vertical discontinuity between lanes 11 and 12.
- Similarities were noted between Fig 8a of this article [1] and Fig 2 of a *J*. *Biol Chem*. article [2]. *PLOS ONE* Fig 8a alpha Tubulin lanes 2–7 appear similar to *J*. *Biol Chem*. Fig 2a alpha-Tubulin lanes 1–6 in the opposite orientation; and *PLOS ONE* Fig 8a alpha Tubulin lanes 8–13 appear similar to *J*. *Biol Chem*. Fig 2c VEGFR-1 lanes 1–6 in the opposite orientation.
- Fig 9b: A vertical discontinuity was noted between lanes 5 and 6 of the Occludin blot. In addition, differences in band patterns between the Occludin and a-Tubulin data call into question whether they were obtained using the same blot.

The authors noted that an incorrect representative confocal microscopy image was used in Fig 4 and they provided a replacement image for the VEGF A 24hr panel. The authors also provided further raw data underlying Fig 7, 8 and 9. The authors explained that blots provided for Fig 7 are representative of several repeated experiments, but these were not used in the quantification of bands which were performed on the raw blot images the authors provided post-publication. The images provided for Figs 8 and 9 were not sufficient to resolve all of the image concerns outlined above.

The authors acknowledged the similarity between Fig 8 of the *PLOS ONE* article [1] and Fig 2 of the *J*. *Biol Chem* article [2] but are unable to explain how this may have occurred.

In light of these overall concerns, which call into question the reliability of the published data, the *PLOS ONE* Editors retract this article.

XQ, LS, SLC, DA, AA, MBG, MEB agreed with the retraction. LW, SC, WGJ did not respond. JC did not agree with retraction. SAV is deceased.
